# The immunological and clinical effects of mutated ras peptide vaccine in combination with IL-2, GM-CSF, or both in patients with solid tumors

**DOI:** 10.1186/1479-5876-12-55

**Published:** 2014-02-24

**Authors:** Osama E Rahma, J Michael Hamilton, Malgorzata Wojtowicz, Omar Dakheel, Sarah Bernstein, David J Liewehr, Seth M Steinberg, Samir N Khleif

**Affiliations:** 1 Cancer Vaccine Branch, CCR, NCI, 10 Center Drive, Bethesda, MD 20892, USA; 2 OD, CCR, NCI, 9030 Old Georgetown Rd, Bethesda, MD 20892, USA; 3 LUACR, DCP, NCI, 9609 Medical Center Drive, Rockville, MD 20850, USA; 4 Walter Reed National Military Medical Center, 8901 Wisconsin Ave, Bethesda, MD 20814, USA; 5 Biostatistics and Data Management Section, CCR, NCI, 9609 Medical Center Drive, Rockville, MD 20850, USA; 6 Georgia Regents University Cancer Center, 1411 Laney Walker Blvd, Augusta, GA 30912, USA; 7 University of Virginia, Charlottesville, VA 22908, USA

**Keywords:** Ras, Peptide, Vaccine, IL-2, GM-CSF, Immune response

## Abstract

**Background:**

Mutant *Ras* oncogenes produce proteins that are unique to cancer cells and represent attractive targets for vaccine therapy. We have shown previously that vaccinating cancer patients with mutant ras peptides is feasible and capable of inducing a specific immune response against the relevant mutant proteins. Here, we tested the mutant ras peptide vaccine administered in combination with low dose interleukin-2 (IL-2) or/and granulocyte-macrophage colony-stimulating factor (GM-CSF) in order to enhance the vaccine immune response.

**Methods:**

5000μg of the corresponding mutant ras peptide was given subcutaneously (SQ) along with IL-2 (Arm A), GM-CSF (Arm B) or both (Arm C). IL-2 was given SQ at 6.0 million IU/m^2^/day starting at day 5, 5 days/week for 2 weeks. GM-CSF was given SQ in a dose of 100μg/day one day prior to each ras peptide vaccination for 4 days. Vaccines were repeated every 5 weeks on arm A and C, and every 4 weeks on arm B, for a maximum of 15 cycles or until disease progression.

**Results:**

We treated 53 advanced cancer patients (38 with colorectal, 11 with pancreatic, 1 with common bile duct and 3 with lung) on 3 different arms (16 on arm A, 18 on arm B, and 19 on arm C). The median progression free survival (PFS) and overall survival (OS) was 3.6 and 16.9 months, respectively, for all patients evaluable for clinical response (n = 48). There was no difference in PFS or OS between the three arms (*P =* 0.73 and 0.99, respectively). Most adverse events were grade 1-2 toxicities and resolved spontaneously. The vaccine induced an immune response to the relevant ras peptide in a total of 20 out of 37 evaluable patients (54%) by ELISPOT, proliferative assay, or both. While 92.3% of patients on arm B had a positive immune response, only 31% of patients on arm A and 36% of patients on arm C had positive immune responses (*P* = 0.003, Fisher’s exact test).

**Conclusions:**

The reported data showed that IL-2 might have a negative effect on the specific immune response induced by the relevant mutant ras vaccine in patients with advanced cancer. This observation deserves further investigations.

**Trial registration:**

NCI97C0141

## Background

*Ras* oncogenes are extensively characterized mutated genes in human cancers [1,2]. With a single amino acid substitution, the ras protein can potentiate transforming capabilities in human cells [3]. Such point mutated *Ras* genes have been found in a broad spectrum of human malignancies, notably at codons 12, 13, and 61 [4]. Codon 12 mutations account for more than 90% of all *Ras* mutations in human cancers [5]. *Ras* mutations are prevalent in many types of tumors including pancreatic (90%) [6], colorectal (50%) [7] and lung cancer (30%) [8]. Mutant ras peptides are processed and presented as foreign antigens by both MHC class I or II molecules [9,10]. The products of mutant ras antigens represent attractive targets for therapeutic cancer vaccines due to their distinctive expression in tumor tissues as compared to normal tissues. We and others have shown that vaccinating patients with mutant ras peptides could elicit specific immune responses against the corresponding antigens [11-14]. In a previously reported phase I clinical trial, we demonstrated the safety of vaccinating advanced cancer patients with the corresponding mutated ras peptides [12]. In another study where patients were vaccinated in the adjuvant setting, the corresponding mutated ras vaccines were capable of generating specific immune responses with encouraging clinical outcomes in colorectal and pancreatic cancer patients [15]. Therefore, in an attempt to enhance the immune response generated with our mutated ras peptide vaccine, we conducted the current study where we combined this vaccine with interleukin-2 (IL-2), granulocyte-macrophage colony-stimulating factor (GM-CSF) or both. This is with the hope that the enhanced vaccine-induced immune response may translate to an improved clinical efficacy.

IL-2 plays a major role in enhancing the cytolytic activity of T lymphocytes [16,17]. In addition, many investigators have shown that IL-2 can improve the immune effect of cancer vaccines by potentiating the effect of tumor-specific lymphocytes [18-20]. Based on this evidence, we used low dose subcutaneous (SQ) IL-2 along with the mutant ras peptide vaccine on one arm of the study. GM-CSF is known to be an important element in stimulating the growth of the antigen presenting cells such as dendritic cells (DCs) [21]. In addition, GM-CSF has been found to enhance the vaccine efficacy by increasing the number of immature DCs (iDCs) at the vaccination site [22] and enhancing their maturation and migration [23]. Accordingly, we used GM-CSF SQ along with the ras vaccine in the second arm (arm B) of this trial. Finally, patients in the third study arm (arm C) received the ras vaccine in combination with both GM-CSF and low dose SQ IL-2, which was supported by our pre-clinical data showing a synergistic effect of this combination by inducing a larger number of cytotoxic T lymphocytes (CTLs) and a greater cytokine release response [24].

## Methods

### Study objectives

The primary endpoint of this pilot study was to evaluate the immune response generated with our ras peptide vaccine admixed with Detox TM PC adjuvant when administered with IL-2, GM-CSF or the combination of both (IL-2 and GM-CSF). The secondary objectives were to evaluate toxicities observed on each treatment arm, and to explore clinical responses noted with our vaccination strategy.

### Patient selection

Patients were assigned to three groups. All groups received tumor-specific mutated ras peptide vaccine with Detox™ PC admixture. The vaccine was given in combination with Il-2 (SQ) in arm A, GM-CSF (SQ) in arm B, and both Il-2 and GM-CSF (SQ) in arm C. All study patients had histologically proven advanced solid tumors expressing different *Ras* mutations and received multiple lines of therapy. All enrolled patients met the protocol eligibility criteria, including ECOG performance status of 0-1 and life expectancy of more than 3 months. The main exclusion criteria included evidence of brain metastasis, history of autoimmune disease, and history of other malignancies except basal carcinoma of the skin. Both the National Cancer Institute (NCI) and National Naval Medical Center (NNMC) Institutional Review Boards (IRBs) approved the protocol, and the patients’ consent was obtained prior to enrollment.

### Peptide selection

The peptides used in this study were 13-mer peptides (residues 5-17) corresponding to the tumor *Ras* mutations (Table 1). The *Ras* DNA mutations were determined by Restriction Fragment Length Polymorphism (RFLP) and/or sequencing analysis of PCR amplified DNA extracted from paraffin embedded tumor and/or fresh tumor biopsy.

**Table 1:** Ras peptides used for vaccination

**Ras peptides**	**Amino acid sequence**	**Vaccinated patients by arm**
	**5 6 7 8 9 10 11 12 13 14 15 16 17**	
**ras 5-17**	Lys- Leu-Val- Val- Val- Gly- Ala- Gly- Gly- Val- Gly- Lys- Ser	Nome
**(wild type)**		
**ras 5-17**	Lys- Leu-Val- Val- Val- Gly- Ala- **Asp**- Gly- Val- Gly- Lys- Ser	A: 1-4, 9-12, 14, 16
**(Gly→Asp)**		B: 3-6, 8-10, 14-16
		C: 1-5, 7, 9-11, 13-19
**ras 5-17**	Lys- Leu-Val- Val- Val- Gly- Ala- **Val**- Gly- Val- Gly- Lys- Ser	A: 5, 6, 15
**(Gly→Val)**		B: 1, 7, 11, 12, 17, 18
		C: 6, 12
**ras 5-17**	Lys- Leu-Val- Val- Val- Gly- Ala- **Cys**- Gly- Val- Gly- Lys- Ser	A: 7, 8, 13
**(Gly→Cys)**		B: 2, 13
		C: 8

The peptides used in the study were 13-mer peptides (residues 5-17) corresponding to the tumor *Ras* mutations. Corresponding mutant part of the peptide is bolded.

### Peptide manufacturing and vaccine preparation

Synthesis of the peptides was done under contract with Multiple Peptide Systems (San Diego, CA) for clinical use. The vaccine contained the peptide and Detox™ PC as an adjuvant. The vaccine was prepared in the following manner: the pharmacist calculated the volume corresponding to 120% of the dose of ras peptide. Sterile water (0.48 ml) was then added to a vial containing 0.12 ml Detox PC to bring the final vaccine volume to 0.6 ml. The sterile water/Detox PC emulsion was shaken. The appropriate ras peptide was added to the resulting emulsion. The final admixture (1.8 ml) was gently vortexed (not shaken) for 15-30 seconds. The product was labeled with the peptide, concentration, time of preparation, and “one hour expiration”. 1.5 ml of the admixture was used for the patient’s vaccination.

### Vaccine administration

All study patients were assigned to one of three treatment arms. Patients in all treatment groups received specific mutated ras peptide vaccine with admixture of Detox™ PC adjuvant administered SQ. In addition, arm A patients received IL-2 (SQ), arm B patients received GM-CSF (SQ), and arm C patients received both IL-2 and GM-CSF (SQ).

All patients received 1.5 ml of the vaccine solution with one half of the total dose administered into each of two sites, over the deltoids, the thighs, or the abdomen (0.75 ml/site). Vaccination was repeated every 5 weeks on arm A and C, and every 4 weeks on arm B for a total of 3 vaccinations. Patients continued to receive sets of 3 additional vaccinations for a total of 15 vaccinations as long as they demonstrated lack of disease progression. After receiving the vaccine, patients were observed in the outpatient clinic for any acute hypersensitivity reaction. IL-2 was administered SQ in a dose of 6.0 million IU/m^2^/day starting 4 days after vaccination, 5 days/week for 2 weeks in the arms, abdomen, or thighs. The initial IL-2 dose was administered in the outpatient clinic with subsequent injections were done via self-administration by the patient on an outpatient basis. GM-CSF was given SQ starting one day prior to the vaccination and continued for 4 days at a dose of 100μg/day. GM-CSF was administered at two separate sites, 50μg per site, at the same sites of the vaccine injection. The GM-CSF was administered in the outpatient clinic directly after the peptide injection but with a separate syringe. Patients were monitored for any hypersensitivity reaction.

### Clinical monitoring

Patients were evaluated for toxicity and tumor response during treatment. Tumor response was assessed by physical exam and CT scan according to RECIST criteria at baseline, after each set of 3 vaccinations, and every 2 months during follow-up. Patients were taken off study due to either deterioration in performance status, disease progression or request to withdraw from the study. Disease progression was defined according to the modified WHO criteria of progression as the appearance of new lesions and/or a 25% increase of measurable lesions as evident by CT scan. Once patients had progressed, follow-up was not required except to document late toxicities and death. Adverse events and toxicities were defined and graded according to the NCI Common Toxicity Criteria.

### Immune monitoring

Peripheral blood mononuclear cells (PBMCs) were collected within 1 hour prior to every vaccination and every 2 months during follow-up. PBMCs were isolated from heparinized venous blood by Ficoll Hypaque centrifugation, washed, and cryopreserved in 2-mL vials, using a CryoMed freezer. The immunological response was assessed by *in vitro* T cell proliferation assay and enzyme-linked immunosorbent spot (ELISPOT) assay before and after each vaccination.

### In vitro T cell proliferation assay

The patient’ PBMCs were incubated *in vitro* with the appropriate tumor-specific ras peptide and evaluated for peptide-induced proliferation following up to 5 days of incubation. Cells were pulsed with [3H]-thymidine for the final 18-24 hours of their culture. Proliferation was measured and quantified by the incorporation of [3H]-thymidine. Positive control included cells pulsed with a recall antigen peptide (influenza matrix 58-66, GILGFVFTL). A proliferation of more than two fold above the control of the wild-type ras peptide at 2 time points was considered as a positive response.

### ELISPOT assay

All ELISPOT assays were performed at the Laboratory of Cell-Mediated Immunity, SAIC-Frederick (CLIA-certified lab). Two frozen normal donor controls with known responsive values were run with each assay to assure quality control of the assay results. For all assays, at least one of the two controls was within 2 standard deviations of the laboratory-generated means for CMV and CEF. All assays were performed on 7-8 day *in vitro* stimulated PBMCs (100K/well) as the effectors and peptide-pulsed autologous PBMCs (100K/well) as the antigen presenting cells (APCs). When possible, PBMCs from the earliest time point were used as the APCs. However, if this was not possible, the pulsed PBMCs were assayed alone to make sure they were not producing any spots. Briefly, the day before assay setup, 96-well polyvinylidene fluoride (PVDF) membrane, HTS opaque plates (Millipore, Billerica, Massachusetts, MSIPS4W10) were coated overnight with a 1:100 dilution of anti-human IFN-γ capture antibody (1mg/mL, Mabtech Inc., Mariemont, OH, Cat# 3420-3-1000) in Dulbecco's phosphate buffered saline (DPBS) at room temperature. Antibody-coated plates were washed four times in DPBS the next day and blocked with 5% human AB ELISPOT medium at 37°C for approximately 2 hours. 1 × 10^5^*in vitro* stimulated PBMCs and 1 × 10^5^ autologous, peptide-pulsed PBMCs were plated per well. The plates were incubated for 18-20 hours at 37°C. The next day, the plates were manually washed six times with 0.05% Tween 20 in DPBS, followed by a 2-hour incubation at room temperature with a 1:2000 dilution of the biotinylated secondary antibody, anti-human IFN-γ (1 mg/mL Mabtech Inc., Mariemont, OH, Cat# 3420-6-1000) in DPBS/1% bovine serum albumin/0.05% Tween. After incubation and four washes in DPBS to remove excess antibody, a 1:3000 dilution of streptavidin alkaline phosphatase (Mabtech, Mariemont, OH, Cat# 3310-10) in DPBS/1% bovine serum albumin, was added to each well for 1 hour at room temperature followed by 4 manual washes in DPBS. Finally, the BCIP/NPT substrate, 100 μl/well, (KPL, Gaithersburg, Maryland, Cat# 50-81-08) was added for 7-10 minutes, resulting in the development of spots. The reaction was stopped by washing three times in distilled water. Plates were dried overnight and the spots were visualized and counted using the ImmunoSpot Imaging Analyzer system (Cellular Technology Ltd., Cleveland, OH). ELISPOT results were expressed as the “number of spots per 10^6^ responder cells” after subtracting background spots obtained in wells of effectors with non-pulsed PBMCs. For each subject, PBMCs obtained before and after vaccination were analyzed in the same assay to avoid inter-assay variability. An increase of number of spots to more than two fold above the control of the wild-type ras peptide or the irrelevant TAX peptide at 2 time points was considered as a positive response.

### Regulatory T cells (T-regs)

Cryopreserved PBMCs were thawed rapidly at 37°C. The cells were transferred into 15 mL conical tubes (Corning, Lowell, MA) and diluted to 10 mL by dropwise addition of RPMI medium containing 20% FBS. The cells were pelleted by low-speed centrifugation at 250 × g for 10 min at 25°C. Supernatants were discarded and cell pellets resuspended in 5 mL of DPBS containing 2% (huAB) serum to block cell surface Fc receptors. The samples were mixed briefly and incubated on ice for 15 minutes. Following incubation the cells were pelleted by centrifugation as described before, washed two times with DPBS containing 2% bovine serum albumin (BSA; DPBS/2% BSA) and resuspended in 1 mL of DPBS/2% BSA. The cells were counted in a Coulter counter and adjusted to a final concentration of 10 × 10^6^/mL in DPBS/2% BSA. The cells (1 × 10^6^/tube) were stained for surface markers (CD25, CD3, and CD4) for 20 minutes at room temperature (RT) in the dark and washed two times with DPBS/2% BSA. Intracellular staining for FoxP3 was carried out using human FoxP3 buffer prepared as described by the manufacturer (BD BioSciences, San Jose, CA). Briefly, following staining of surface antigens, cells were resuspended in 2 mL of fixing solution (buffer A) and incubated for 10 minutes at RT in the dark. Cells were washed two times with PBS/2% BSA, resuspended in 0.5 mL permeabilization solution (buffer C) and incubated for 30 minutes at RT in the dark. Cells were washed two times in PBS/2% BSA and stained with anti-human FoxP3 antibody for 30 minutes at RT in the dark. Cells were then washed two times and resuspended in 0.5 mL of PBS/2% BSA for four-color flow cytometric analysis using the FACSCanto cytometer (BD BioSciences, San Jose, CA) running FACS Diva acquisition software (version 6.0). Each assay contained a parallel set of cells stained with relevant isotype controls (Alexa Fluor 488 IgG1 and PE IgG1). Flow cytometric data analysis was carried out using FlowJo Software. T cells were identified by plotting CD3 by side scatter. CD4+ T cells were identified by further gating the CD3+ subset by forward and side scatter and by CD4. The regulatory CD4+ T cell subset was identified by plotting CD25 versus FoxP3 with the quadstat setting determined based on the isotype control tube. The quadrant markers of the CD25 versus FoxP3 dot plot were set based on the isotype controls. In each case the pre-vaccination sample and the post 4 or 8-vaccination sample (based on how many vaccines the patient received) were tested side by side in the same experiment and were done from frozen samples. This testing strategy was used to minimize variability from day to day in staining or thawing. The samples were tested in 4 independent setups over 3 days. We have included 2 internal controls in each experiment, one of those being a frozen leukapheresis sample that was included in each test run as a measure of interassay reproducibility.

### Statistical analysis

Actuarial analyses were performed on the survival and progression free survival (PFS) data, starting at the on-study date, or the date of progression when determining probability of survival following progression, using the Kaplan-Meier method. Curves were compared using the log-rank test. Survival and PFS times were censored if the patient was alive and/or without progression as appropriate at the last follow-up date. The fractions of patients with immune responses on the three arms were compared using Mehta’s modification to Fisher’s exact test [25]. All reported *P* values are two-tailed, and unadjusted for multiple comparisons.

## Results

### Patient profiles

Fifty-three patients were enrolled on this trial (16 on arm A, 18 on arm B, and 19 on arm C). Patient characteristics are summarized in Table 2, Table 3, Table 4. Age of the patients ranged from 33 to 79 years with a mean of 55.3 years (standard deviation of 10.7 years). The majority of the patients, 38 out of the 53 enrolled, had colorectal cancers (11 on arm A, 12 on arm B and 15 on arm C), eleven patients had pancreatic cancer (3 on arm A, 5 on arm B and 3 on arm C), three patients had lung cancer (2 on arm A and 1 on arm C), and one patient on arm B had cancer of the common bile duct. All patients had adenocarcinoma except for one who had poorly differentiated non-small cell carcinoma of the lung (3C). All patients’ tumors harbored *Ras* mutations as follows: the majority of patients (36 patients) had a glycine to aspartic acid substitution and the rest had either a glycine to valine (11 patients) or a glycine to cysteine (6 patients) substitution. All patients were heavily pretreated. Most underwent at least one surgical resection except for two patients on each arm (5A, 6A, 4B, 18B, 3C, 19C). At least one chemotherapy regimen was administered to all patients except for one (16C). In addition, 39 of the 53 patients received 2 or more chemotherapy regimens prior to enrollment on the protocol. A total of 21 patients received radiation therapy. Twelve patients were enrolled on the trial with no evidence of disease following resection of their primary or metastatic disease: 5 patients on arm A (7A, 9A, 11A, 12A, 14A), 3 patients on arm B (1B, 4B, 14B) and 4 patients on arm C (2C, 11C, 13C, 15C) (Table 2, Table 3, Table 4).

**Table 2:** Arm A (vaccine + IL-2): patient profiles, clinical, and immunological outcomes

**Pt**	**Age**	**Cancer**	**Stage on enrollment**	**# of cycles**	**Off-study reason**	**PFS**^**#**^**(ms)**	OS* (ms)	**Immune response**			
								**Proliferative assay**		**Elispot**	
								**Pre-vaccine**	**Post-vaccine**	**Pre-vaccine**	**Post-vaccine**
1A	56	Colon	IV	2	PD	0.5	5.5	ND			
2A	57	Colon	IV	3	PD	3.9	16.8	NA		-	-
3A	62	Colon	IV	3	PD	5.8	21.5	-	-	-	-
4A	50	Pancreatic	IV	3	PD	3.6	6.2	-	-	-	-
5A	60	Lung	IV	1	PD	1	2.8	ND			
6A	59	Colon	IV	3	PD	3.5	8.9	NA			
7A	52	Colon	NED	11	Completed	129+	129+	-	+	-	+
8A	68	Lung	IV	3	PD	3.6	13.1	-	-	-	-
9A	56	Colon	NED	10	PPS	18.8	37.2	-	+	-	+
10A	63	Colon	IV	3	PD	3.3	19.9	-	-	-	-
11A	42	Pancreatic	NED	6	PD	7.5	24.1	-	-	-	-
12A	39	Colon	NED	3	PD	6.2	23	-	-	-	-
13A	67	Colon	IV	3	PD	3.5	4.8	-	+	+	+
14A	51	Colon	NED	3	PD	7.1	41.3	-	-	-	-
15A	60	Pancreatic	IV	3	PPS/Lost to follow-up	2.7+	5.3	NA		-	-
16A	61	Colon	IV	3	PD	3.6	17.3	-	+	-	-

^#^Progression free survival was calculated as time from the date the consent was signed until evidence of disease progression or last follow-up without progression (+). *Overall Survival was calculated as time from consent date until death or last follow-up (+). ND, not done because patient received 2 or less vaccines; NA, sample not available. Immune response was marked as negative (-) or positive (+) as described in the manuscript.

*Abbreviations*: *NED* No Evidence of Disease, *PD* Progression of Disease, *PPS* Poor Performance Status, *SD* Stable Disease, *PFS* Progression Free Survival, *OS* Overall Survival, *ms* Months.

**Table 3:** Arm B (vaccine + GM-CSF): patient profiles, clinical data, and immunological outcomes

**Pt**	**Age**	**Cancer**	**Stage on enrollment**	**# of cycles**	**Off-study reason**	**PFS**^**#**^**(ms)**	OS* (ms)	**Immune response**			
								**Proliferative assay**		**Elispot**	
								**Pre-vaccine**	**Post-vaccine**	**Pre-vaccine**	**Post-vaccine**
1B	55	Biliary	NED	3	PD	2.9	8.7	+	+	+	+
2B	33	Colon	IV	3	PD	2.9	16.9	-	+	NA	
3B	51	Colon	IV	5	PPS/Lost to follow-up	9.2+	52.6	+	+	-	+
4B	48	Pancreatic	NED	13	Completed	35.4+	35.4	-	+	-	-
5B	51	Colon	IV	1	PD	1	1.5	ND			
6B	38	Colon	IV	1	Refused further Tx	0.2+	8.3	ND			
7B	60	Pancreatic	IV	3	PD	4.6	21	-	-	NA	
8B	57	Colon	IV	3	PD	3.1	6.8	NA			
9B	52	Colon	IV	3	PD	3.3	28.3	-	+	+	+
10B	58	Pancreatic	IV	3	PD	2.9	20.9	+	+	-	+
11B	45	Pancreatic	IV	2	PD	1	7.3	ND			
12B	79	Colon	IV	3	PPS	12.9	42.1	-	+	-	+
13B	43	Colon	IV	3	PD	2.6	6.3	-	-	-	+
14B	78	Colon	NED	8	Left voluntarily	16.8	69	-	+	-	+
15B	63	Colon	IV	3	PD	2.8	11.6	+	+	-	+
16B	64	Colon	IV	6	PD	5.7	20.7	-	+	-	-
17B	71	Colon	IV	6	PD	5.6	25.9	-	+	-	+
18B	58	Pancreatic	IV	2	PD	2.6	3.3	ND			

^#^Progression free survival was calculated as time from the date the consent was signed until evidence of disease progression or last follow-up without progression (+). *Overall Survival was calculated as time from consent date until death or last follow-up (+). ND, not done because patient received 2 or less vaccines; NA, sample not available. Arm B (Vaccine + GM-CSF): Patient Profiles, Clinical Data, and Immunological Outcomes.

*Abbreviations*: *NED* No Evidence of Disease, *PD* Progression of Disease, *PPS* Poor Performance Status, *SD* Stable Disease, *PFS* Progression Free Survival, *OS* Overall Survival, *ms* Months.

**Table 4:** Arm C (vaccine + IL-2 + GM-CSF): patient profiles, clinical data, and immunological outcomes

**Pt**	**Age**	**Cancer**	**Stage on enrollment**	**# of cycles**	**Off-study reason**	**PFS**^**#**^**(ms)**	OS* (ms)	**Immune response**	
								**Elispot**	
								**Pre-vaccine**	**Post-vaccine**
1C	40	Rectal	IV	15	PD	26.5	80.4	NA	+
2C	59	Colon	NED	14	Completed	110.2+	110.2+	-	-
3C	58	Lung	III	1	PPS	8	72.8	ND	
4C	35	Colon	IV	6	PD	6.9	18.7	-	-
5C	52	Rectal	IV	3	PD	3.2	6.4	-	-
6C	36	Rectal	IV	2	PPS/Lost to follow-up	2+	9.2	ND	
7C	75	Colon	IV	1	PD	0.9	5.2	ND	
8C	49	Colon	IV	3	PD	3.5	5.5	-	-
9C	42	Colon	IV	3	PD	4	7.6	-	-
10C	48	Colon	IV	2	PD	1.7	4.7	ND	
11C	48	Rectal	NED	15	Completed	120.4+	120.4+	-	+
12C	54	Colon	IV	3	PD	3.6	26.6	+	+
13C	57	Colon	NED	3	PD	3.6	9.1	NA	
14C	66	Pancreatic	IV	2	PD	2.4	2.8	ND	
15C	56	Rectal	NED	4	PD	3.6	28.5	-	+
16C	73	Pancreatic	IV	3	PD	3.6	6.6	-	-
17C	51	Colon	IV	2	PD	1.9	7.9	ND	
18C	57	Colon	IV	3	PPS	4.4	9.8	-	-
19C	66	Pancreatic	IV	2	PD	2.1	2.7	ND	

#Progression free survival was calculated as time from the date the consent was signed until evidence of disease progression or last follow-up without progression (+). *Overall Survival was calculated as time from consent date until death or last follow-up (+). ND, not done because patient received 2 or less vaccines; NA, sample no available. Immune response was marked as negative (-) or positive (+) as described in the manuscript.

*Abbreviations*: *NED* No Evidence of Disease, *PD* Progression of Disease, *PPS* Poor Performance Status, *SD* Stable Disease, *PFS* Progression Free Survival, *OS* Overall Survival, *ms* Months.

### Safety and toxicity

The vaccine was well tolerated for all arms. The majority of toxicities were grade 1 or 2, with fatigue, fever and local site reaction being the most common, accounting for 77%, 71%, and 69% of patients, respectively. The most common treatment-related grade 3 toxicities were fatigue (5.7%), followed by diarrhea (3.8%), vomiting (3.8%), and transaminitis (3.8%) (Table 5). The majority of these toxicities were determined to be related to IL-2 or GM-CSF and resulted in dose reduction in some cases. IL-2 was dose reduced to 50% in 3 patients, 1 patient on arm A (7A) and 2 patients on arm C (5C, 6C). On the other hand, only two patients had 50% dose reduction for GM-CSF, both on arm B (7B, 9B). Grade 4 treatment-related toxicity occurred in only one patient (3A) who developed myocardial infarction that was determined to be possibly related to IL-2. Treatment was discontinued for that patient.

**Table 5:** Grade 3-4 vaccine-related toxicities

**Toxicity grade 3-4**	**Number of patients full cohort**	**Number of patients arm A**	**Number of patients arm B**	**Number of patients arm C**
Fatigue	3	2	0	1
Diarrhea	2	0	0	2
Vomiting	2	0	1	1
Increased transaminase	2	0	1	1
Local site reaction	1	0	1	0
Fever	1	0	0	1
Myalgia	1	0	0	1
Generalized rash	1	0	1	0
Myocardial infarction*	1	1	0	0

*Grade 4 Toxicity.

All grade 3 toxicities were reported for the full cohort and per arm. Only one grade 4 toxicity of myocardial infarction was reported in one patient (3A).

### Clinical response

**Figure 1:**
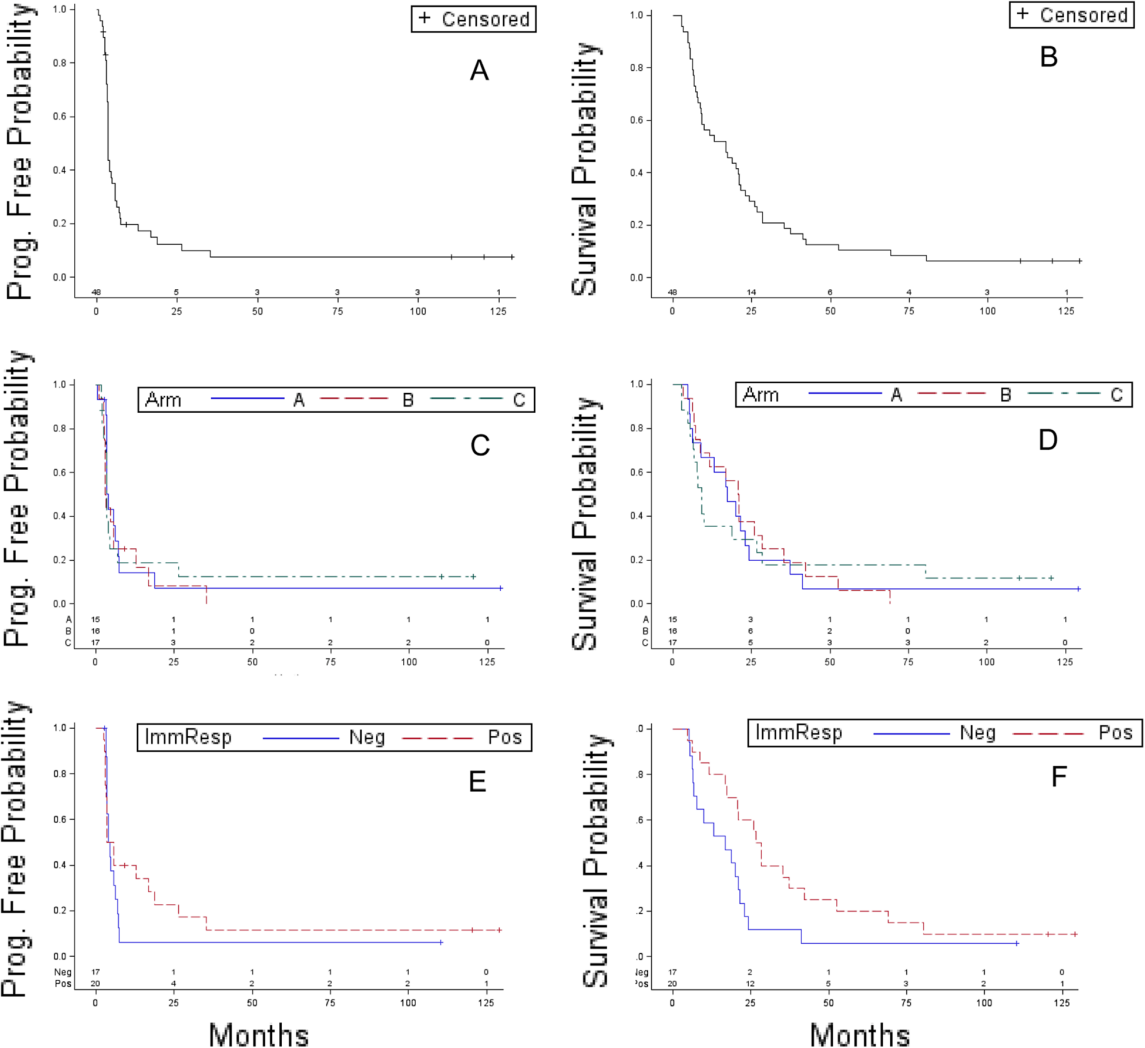
**Clinical outcome.** Kaplan-Meier curves for progression free survival (PFS) and overall survival (OS) for the full cohort **(A, B)**, per arm **(C, D)**, and based on immune response **(E, F)**.

Five patients were excluded from the clinical response analyses since they received less than 2 vaccines. They were removed from the study due to early disease progression (5A, 5B, and 7C), poor performance status (3C), or refusal of further treatment (6B). Therefore, the clinical response was evaluable in only 48 of the 53 treated patients. Of these, 37 had progression of disease during the course of treatment (12 patients on each of arm A and B, and 13 on arm C). Five patients had stable disease (one patient on arm A and 2 patients on each of arm B and C). Six patients remained with no evidence of disease (2 on each arm); interestingly, 4 out of these 6 patients completed the study after they received 11-15 vaccines (7A, 4B, 2C, 11C) (Table 2, Table 3, Table 4). For the full cohort (n = 48), the median progression free survival (PFS) and overall survival (OS) was 3.6 and 16.9 months, respectively (Figure 1). Patients on arm A had a median PFS and OS of 3.9 and 17.3 months, respectively. Patients on arm B had a median PFS and OS of 3.2 and 20.8 months, respectively, and patients on arm C had a median PFS and OS of 3.6 and 9.1 months, respectively (Figure 1). The difference in PFS or OS between arms A, B and C was not statistically significant (*P =* 0.74 and 0.99, respectively). Finally, we conducted an unplanned subgroup analysis for patients with stage IV advanced colorectal cancer (n = 26) since they represent the majority of patients treated on the three arms. We found that patients with advanced colorectal cancer had a median PFS and OS of 3.5 and 14.2 months, respectively.

### Immunologic data

#### Immune response assays

**Figure 2:**
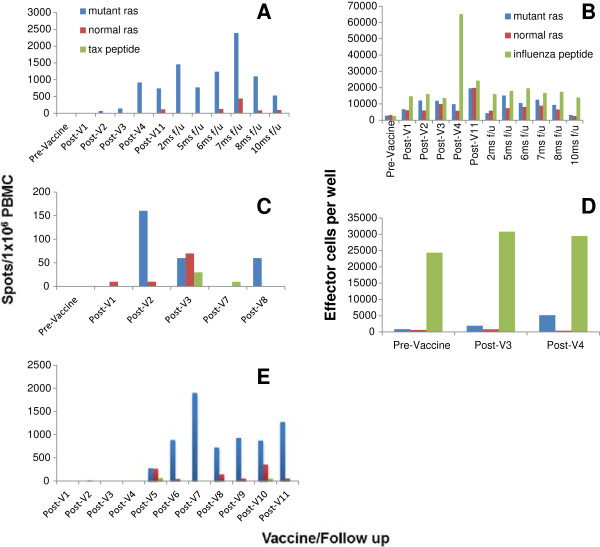
**Immune responses measured by ELISPOT assay and *In vitro *T cell proliferation assay.** ELISPOT results for patient 7A **(panel A)**, 14 B **(panel C)**, and 11C **(panel E)** who had positive immune responses to the mutant ras peptide in blue compared with the normal ras peptide in red and the control peptide (tax) in green. Panels **B** and **D** show positive T cell proliferative assay responses for patient 7A and 14B, respectively in blue compared with the normal ras peptide in red and the positive control peptide (influenza) in green. Abbreviations: Pre-vaccine, Pre-vaccination sample; Post-V, Post-vaccination sample marked by the vaccine number; f/u, Follow up sample marked in months (ms) from the last post vaccine sample.

Immune assays were planned to be performed only in patients who received 3 vaccines or more, a total of 40 patients (14 on each of arm A and B, and 12 on Arm C). However, blood samples were not available in 3 out of these 40 patients (one on each arm: 6A, 8B, and 13C). Therefore, immune responses were measured in a total of 37 patients (13 on each of arm A and B, and 11 on arm C). Immune responses were measured using either ELISPOT, proliferative assay, or both in Arms A and B, and ELISPOT only in Arm C given the limited sample availability (Table 2, Table 3, Table 4). Immune response was considered positive if either one of the performed assays was positive. Only 7 out of 37 patients (19%) demonstrated a baseline endogenous immune response to the corresponding ras peptide by ELISPOT, proliferative assay, or both (1 on each of arm A and C and 5 on arm B). Overall, a total of 20 out of 37 evaluable patients (54%) had positive immune responses by ELISPOT, proliferative assay, or both. There were significant differences in immune responses generated on each arm (*P =0.003, Mehta’s modification to Fishers exact test*). While 12 out of 13 evaluable patients (92.3%) on arm B had positive immune responses, only 4 out of 13 evaluable patients (31%) on arm A and 4 out of 11 evaluable patients (36%) on arm C had positive immune responses (Table 2, Table 3, Table 4). Interestingly, the positive ELISPOT responses were associated with positive T cell proliferation responses in all patients evaluated by both assays except one (13B); on the other hand, 3 patients with positive T cell proliferation responses had negative ELISPOT responses (16A, 4B, and 16B). Such an association could not be ascertained on arm C since only ELISPOT data was available. Figure 2 shows examples of three patients with positive immune response, one on each arm (Patients# 7A, 14B, and 11C).

#### T-regulatory cells (T-reg) analysis

**Figure 3:**
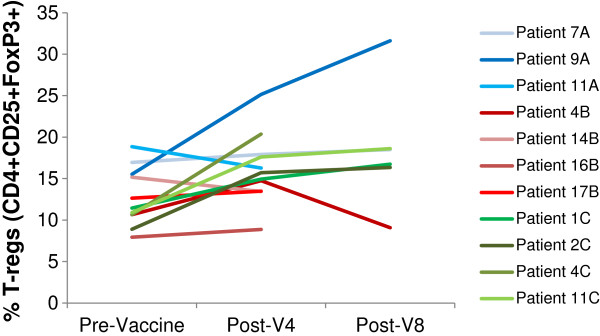
**Regulatory T cells (T-regs).** The percentage of T regulatory cells (CD4 + CD25 + FoxP3+) measured in the peripheral blood of selected patients on Arm A (7A, 9A, 11A) marked in blue colors, B (4B, 14B, 16B, 17B) marked in red colors, and C (1C, 2C, 4C, 11C) marked in green colors. Samples were taken pre-vaccination and post-vaccine #4 and #8.

T-regs (CD4 + CD25 + FoxP3+) were measured in the peripheral blood pre-vaccination and post 4 or 8 vaccinations in 11 patients only, due to the limited availability of blood samples (3 patients on Arm A and 4 patients on each of Arm B and C). An increase in T-regs was defined as an increase in the frequencies of post-vaccination T-regs by at least 25% compared to pre-vaccination. Interestingly, all tested patients on Arm B had a notable increase in T-reg frequencies in the post-vaccination samples compared with pre-vaccination samples, while none of the tested patients on arm C and one out of 3 tested patients on arm A had an increase in T-reg frequencies (Figure 3).

### Clinical and immune response correlation

We performed a survival analysis based on the resulting immune responses. Only patients with available immune response data (n = 37) were included in this analysis, whereas in the previous clinical response analysis there were 48 patients included; thus, 23% of the data were missing in this analysis (11/48). First, we performed this clinical-immune response survival analysis by arm (A (n = 13), B (n = 13), C (n = 11)). We found no evidence for an immune response effect for either OS or PFS, by arm. Second, we performed the same analysis for the full cohort (n = 37). We found a weak immune response effect with respect to OS and no evidence for an immune response effect with respect to PFS for the full cohort. Patients with a positive immune response (n = 20) had a median PFS and OS of 4.6 and 27.5 months, respectively, compared to a median PFS of 4.2 months (*P* = 0.43) and a median OS of 16.8 months for patients with a negative immune response (n = 17, *P =* 0.038) (Figure 1). However, when we performed a stratified test to compare the immune response curves while controlling for the effect of the three arms (n = 37); the *P* values were 0.086 and 0.15 for OS and PFS, respectfully, consistent with no evidence for an immune response effect with respect to OS and PFS.

## Discussion

In this pilot study, we evaluated the feasibility, safety and immune response generated with our mutant ras peptide vaccine when administered in combination with either low dose SQ IL-2, GM-CSF or both cytokines. All vaccine combinations were well tolerated with a comparable safety profile within all treatment arms. 54% of patients generated an immune response against the relative mutated peptide vaccine with a significant difference between the 3 arms; 92% of patients on arm B, 31% on arm A and 36% on arm C had positive immune responses (*P = 0.003*).

The mutant ras peptides have been investigated as cancer vaccines in several clinical trials alone [14,26] or in combination with GM-CSF [13,27,28]. Carbone et al. treated 21 patients who had various stages of colon, pancreatic and lung cancer with 17-mer peptides corresponding to the tumor’s mutation and demonstrated a median OS of 3.8 months and an immune response against the mutant ras peptide in 38% of patients as measured by ELISPOT [14]. Gjertsen et al. vaccinated 38 patients who had advanced pancreatic cancer with a mixture of 4 mutant ras peptides reflecting the substitution of Gly at position 12 with an Asp, Cys, Val or Arg residue in combination with GM-CSF and reported a median OS of 2.7 months and peptide-specific immunity of 58% as measured by DTH and T-cell response [13]. The patient population on these trials is comparable to ours, given their advanced disease and the prior treatment they received. However, the majority of patients on our trial had colorectal cancer and also had a longer period of follow-up (up to 120.4 months). It is difficult to compare the immune response elicited by the ras peptide on this trial to the others due to the variability of immune assays used in these trials and the lack of their validation as a measurement of immune response [29].

Interestingly, all evaluable patients but one developed an immune response against the corresponding ras peptide administered in combination with GM-CFS only in arm B (92%). In contrast, the ras peptide failed to induce an immune response in the majority of patients who received IL-2 with or without GM-CSF (immune response of 31% on arm A and 36% on arm C). We and others have shown that the administration of IL-2 could lead to the expansion of T-regs *in vitro* and *in vivo* and therefore, to the inhibition of an immune response [18,30]. Whether the negative IL-2 effect on immune response in this trial was mediated by T-reg expansion could not be determined due to the limited available samples tested for T-regs.

Although this trial was not designed primarily to test for clinical efficacy, the median PFS and OS for the full cohort (3.6 and 16.9 months, respectively) was encouraging, given the fact that the majority of these patients had advanced disease on enrollment and were heavily pretreated. In addition, in an unplanned subgroup analysis of patients with advanced stage IV colorectal cancer, we found that this heavily pretreated cohort had a median PFS of 3.5 and OS of 14.2 months compared to a historical control of 7.3 and 12.9 months, treated with the best available standard therapy [31]. Interestingly, although patients treated on this trial had a shorter median time to progression, they had a slightly longer median OS compared to historical control. Accordingly, we calculated “the post progression survival (PPS)”, defined as time from progression to death, for patients with advanced stage IV colorectal cancer treated on our trial. We found the median PPS for patients with advanced stage IV colorectal cancer in our trial to be notably longer compared to the historical control (10.3 months *vs.* 5.6 months). This observation correlates with the recently published proposed model by Stein et al., which suggested that tumor growth rate decreased more with vaccine therapy compared to chemotherapy, leading to a longer survival despite an early progression of disease [32]. This is in correspondence with the revised immune RECIST criteria and the recently published “FDA Guidance for Industry Clinical Considerations for Therapeutic Cancer Vaccines” [33,34]. Indeed, the long follow-up time that patients had on this trial (up to 120.4 months) allowed us to perform such analysis, although we realize that our analysis is limited by the small number of patient sample and the various therapies that the patients received post-vaccination. We and others have shown that patients who generate a T-cell immune response are more likely to have longer survival compared to non-immune responders [18,35,36]. In this trial, although we found a weak evidence for an immune response effect with respect to OS (*P* = 0.038), this effect was weaker when the effect of the three arms was taken into consideration (*P* = 0.086). Indeed, the amount of missing immunological data precludes a meaningful conclusion of the association between immunologic and clinical data in this trial.

## Conclusions

In summary, our trial confirmed the feasibility and safety of using mutant ras peptide vaccine as a personalized treatment for patients with advanced cancers. Indeed, these mutant ras vaccines were shown to be capable of generating a specific immune response against the relevant peptide. Further studies are needed to test whether IL-2 is detrimental to cancer vaccines, given the lower immune response rate in patients who received it. Nonetheless, although not powered to test for clinical efficacy, our study finding correlates with the observations of others indicating that cancer vaccines could lead to longer survival, despite early progression of disease.

## Abbreviations

SQ: Subcutaneously; IL-2: Interleukin-2; GM-CSF: Granulocyte-macrophage colony-stimulating factor; PFS: Progression free survival; OS: Overall survival; PPS: Post progression survival; DCs: Dendritic cells; iDCs: Immature dendritic cells; CTLs: Cytotoxic T lymphocytes; NCI: National Cancer Institute; NNMC: National Naval Medical Center; IRB: Institutional Review Board; RFLP: Restriction Fragment Length Polymorphism; ELISPOT: Enzyme-linked immunosorbent spot assay; T-regs: T-regulatory cells.

## Competing interests

The authors declare that they have no competing interest.

## Authors’ contributions

OER collected the data and drafted the manuscript, JMH participated in patient care, evaluated patients for eligibility, safety endpoints, and efficacy including follow-up assessments. MW participated in patient care, evaluated patients for eligibility, safety endpoints, and efficacy including follow-up assessments. OD collected the data, SB participated in patient care, SMS performed the statistical analysis, DJL performed the statistical analysis, SNK conceived of the study and participated in its design and coordination. All authors read and approved the final manuscript.
